# Serum Krebs von den Lungen-6 before treatment predicts the prognosis of lung cancer in Asian populations: a systematic review and meta-analysis

**DOI:** 10.3389/fimmu.2025.1644573

**Published:** 2025-09-17

**Authors:** Hong Huang, Liangyu Fu, Chenye Feng, Jiawei Zhou, Jiahuan Xu, Jianjun Sun, Ying Pan, Delei Kong, Wei Wang

**Affiliations:** ^1^ Department of Pulmonary Critical Care Medicine, The First Hospital of China Medical University, Shenyang, China; ^2^ Department of Respiratory and Critical Care Medicine, Second Affiliated Hospital of Zhejiang University School of Medicine, Hangzhou, China; ^3^ Department of Laboratory Medicine, The First Hospital of China Medical University, Shenyang, China

**Keywords:** lung cancer, Krebs von den Lungen-6, mucin 1, prognosis, biomarker

## Abstract

**Background:**

Up until now, no clear consensus has been reached on the role of serum Krebs von den Lungen-6 (KL-6) levels in predicting survival in patients with lung cancer. This meta-analysis aimed to assess the prognostic value of serum KL-6 levels before treatment in lung cancer.

**Methods:**

PubMed, Web of Science, Embase, and Cochrane Library were searched for relevant studies from inception to June 23, 2025. This study was registered with PROSPERO (CRD42024568549).

**Results:**

Thirteen studies involving 1,723 patients were included in this meta-analysis. High serum KL-6 levels before treatment were associated with shorter progression-free survival (hazard ratio [HR] 1.89, 95% confidence intervals [95% CI]: 1.46–2.44, P<0.001; heterogeneity: I²=6.5%, P = 0.37) and overall survival (OS) (HR 1.76, 95% CI: 1.37–2.26, P<0.001; heterogeneity: I²=51.9%, P = 0.023). Subgroup analysis revealed the significant value of elevated KL-6 level for predicting OS of patients with lung cancer without interstitial lung disease (ILD) but not for those with ILD. The pooled results indicated that OS and progression-free survival were shortened when serum KL-6 level exceeded 500 U/mL. The serum KL-6 level determined using electrochemiluminescence immunoassay had a greater predictive value for OS than that determined using enzyme-linked immunosorbent assay in this study.

**Conclusion:**

Elevated serum KL-6 levels (>500 U/mL) before treatment represent a biomarker for poor prognosis of lung cancer for Asian patients without ILD. However, in patients with pre-existing ILD, these elevated levels are more likely to indicate the severity and activity of the underlying fibrotic lung disease rather than providing independent prognostic information about the cancer itself. Electrochemiluminescence immunoassay was recommended for determining the serum KL-6 level.

**Systematic review registration:**

https://www.crd.york.ac.uk/PROSPERO/, identifier CRD42024568549.

## 1 Introduction

Lung cancer accounted for 12.4% of global cancer cases and 18.7% of cancer deaths in 2022, and it remains the most frequently diagnosed cancer and the leading cause of cancer-related deaths (1). Modern clinical management of lung cancer is influenced by the clinical characteristics, pathological staging, and tumor histology of patients. However, it heavily depends on molecular biomarkers predictive of disease recurrence, progression, and treatment response, which play a vital role in guiding therapeutic decisions. The biomarkers for prognostic monitoring mainly focus on different gene signatures or proteomic detection in tumor tissues (2, 3). However, repeated tissue biopsies for prognostic monitoring are not readily available. The high cost of genomic and proteomic testing limits its clinical application. Therefore, it is urgent to explore new biomarkers that can be detected using simple methods on easily available specimens for monitoring lung cancer prognosis.

Lakshmanan et al. (4) reported that the abnormal expression of mucin 1 (MUC1) in lung cancer cells was closely related to tumor proliferation, invasion, metastasis, and angiogenesis. Krebs von den Lungen-6 (KL-6), which was originally discovered as a lung cancer-associated antigen, is one of the sialylated carbohydrate antigens on the N-terminal domain of MUC1 (5). It is released into the blood in a soluble form when its extracellular domain is lysed by a protease. Shiels et al. (6) confirmed that participants with baseline KL-6 levels in the fourth quartile had 1.6 times higher risks of developing lung cancer than those with levels in the first quartile after adjustment for smoking. This suggests that high serum KL-6 levels are an independent risk factor for lung cancer. Tanaka et al. (7) reported that elevated circulating KL-6 levels before surgery was associated with shorter survival of patients with non-small cell lung cancer (NSCLC) undergoing radical surgery. These results suggest serum KL-6 level as a biomarker for the prognosis of lung cancer. However, Inata et al. (8) did not observe a significant association between serum KL-6 levels before treatment and overall survival (OS) of patients with stage IA-IV adenocarcinoma, and the treatment schemes used in their study were not restricted. These inconsistent results raise questions about the potential of serum KL-6 level as a prognostic biomarker for lung cancer and how treatment regimens and tissue types affect its predictive value. KL-6 has been widely used for the diagnosis and assessment of interstitial lung disease (ILD) (9, 10). KL-6 is a biomarker for ILD. However, its predictive value for lung cancer in patients with ILD has not been established. These questions have not been reported yet. Based on the previous findings, we hypothesized that serum KL-6 levels before treatment have predictive value for the mortality risk of patients with lung cancer. We indexed the literature on PubMed, Web of Science, Embase, and Cochrane Library and performed meta-analysis to ascertain the correlation between serum KL-6 level before treatment and the prognosis for lung cancer.

## 2 Materials and methods

### 2.1 Literature search

The articles we included in the study adhered to the Meta-analysis Of Observational Studies in Epidemiology reporting checklist. This study was registered with PROSPERO (CRD42024568549). Two investigators (Hong Huang and Chenye Feng) independently searched for relevant articles on PubMed, Web of Science, Cochrane Library, and Embase published up to June 23, 2025. The search terms included (“KL-6” or “Krebs von den Lungen-6” or “MUC-1” or “mucin-1”) and (“lung cancer” or “lung carcinoma”) (**Supplementary Table S1**). The reference lists of the retrieved publications were reviewed to identify potentially eligible articles. The search was limited to English publications.

### 2.2 Selection criteria

The articles that met the following criteria were included: (a) primary research enrolling patients with lung cancer and (b) studies evaluating the independent prognostic value of circulating KL-6 levels before treatment for patient survival. Meta-analyses, reviews, and case reports were excluded. The two reviewers independently screened and assessed the studies, and differences in opinion were resolved through discussion.

### 2.3 Data collection

Hong Huang and Liangyu Fu independently extracted the following data using a standard form: population characteristics, study design, treatment regimens, statistical model, data on outcomes, and other research details. Wei Wang was consulted to resolve the disagreements. We contacted the corresponding authors to obtain the missing data when necessary.

### 2.4 Quality assessment

Hong Huang and Liangyu Fu independently performed the quality assessment and discussed the inconsistencies with Wei Wang, who made the final decision. Quality assessment was performed using the Quality in Prognosis Studies tool (11). The assessment items included study participation, study attrition, prognostic factor measurement, outcome measurement, study confounding, statistical analysis, and reporting. Each domain was appraised as “low,” “moderate,” or “high” based on the level of bias.

### 2.5 Statistical analysis

The data were analyzed using Stata version 15.0. We calculated the pooled hazard ratio (HR) for KL-6 level and patient survival. The multivariate-adjusted HRs were extracted for studies that reported both univariate and multivariate results using the Cox proportional hazards regression model. For studies that did not report hazard ratios (HRs) and 95% confidence intervals (CIs) directly, these values were estimated from Kaplan–Meier survival curves using the method described by Tierney et al. (12). The Chi-squared (χ2) and inconsistency (I^2^) tests were used to evaluate the statistical heterogeneity across the articles. P < 0.1 or I^2^ > 50% indicated significant heterogeneity. Galbraith plots were used to explore the sources of heterogeneity among studies (13). Sensitivity analysis was performed by omitting one study at a time to explore the sources of heterogeneity and assess the reliability of the results. Publication bias was assessed using forest plots and Egger’s test. The contour-enhanced funnel plot with the trim and fill method were used to determine whether the source of asymmetry was due to publication bias when the funnel plot was asymmetric (14, 15). P < 0.05 denoted statistical significance.

## 3 Results

### 3.1 Study identification and study characteristics

**Figure 1:**
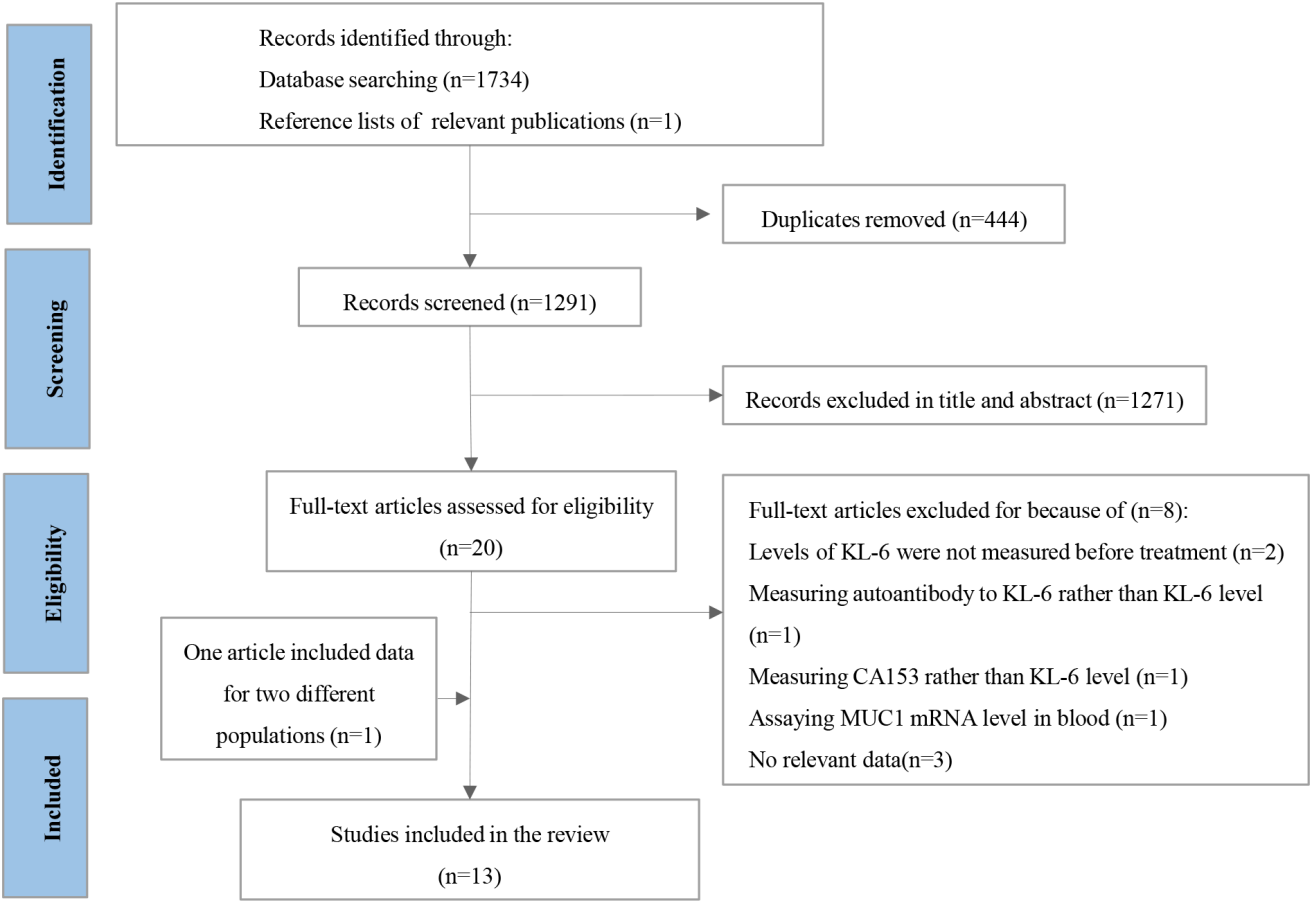
The protocol for searching and selecting studies. CA153, carbohydrate antigen 153; MUC1, mucin 1; KL-6, krebs von den Lungen-6.

A total of 1,735 articles were retrieved. After removing 444 duplicate records, 1,271 studies were excluded based on their titles and abstracts. Eight articles were excluded after careful reading of the full texts. Two studies were excluded because the KL-6 level was not measured before treatment (16, 17). The study by Mitchell et al. (18) was removed because they actually measured carbohydrate antigen 153, another isoform of soluble MUC1. The article of Hirasawa et al. (19) was also excluded because it focused on the prognostic prediction of the natural antibody to KL-6 in patients with lung cancer. The study that assayed MUC1 mRNA levels in the blood for survival was also excluded (20). The other three articles were removed because of insufficient data (21–23). The article by Miyazaki et al. (24) reported survival analyses for two different populations: patients with lung cancer with and without ILD. Therefore, it was split into two independent studies in the subsequent analysis. Thirteen articles (7, 8, 24–33) were included in this meta-analysis. One study analyzed KL-6 level as a continuous variable, and the remaining studies statistically analyzed KL-6 level as a dichotomous variable. The literature screening process is shown in **Figure 1**.

**Table 1** lists the essential characteristics of the included studies. A total of 1,723 patients were included in this study. All studies were from Japan, except for two from Korea (28, 31), and most were retrospective single-center studies. NSCLC was diagnosed in 94.6% of the patients, and 38.1% had stage IV disease. 26.1% of the patients had ILD, except for four studies that did not specify whether the patients had ILD. Curative surgery was performed for patients included in three studies (7, 25, 32). Gefitinib monotherapy was administered to patients in three studies (26, 27, 29). Patients in one study (33) received chemotherapy, and those in another (30) received immune checkpoint inhibitors. The remaining studies involved treatment regimens that included surgery, radiotherapy, chemotherapy, or supportive care. **Table 2** summarizes the characteristics of KL-6 detection. The bias assessments of the included studies are presented in **Supplementary Table S2**. Seven studies had a low overall risk of bias, and the others had a moderate risk.

**Table 1:** Basic characteristics of included studies.

Study	Country	Type of study	Sample size	Type of tumor	Stage	Treatment regimens	Number of patients with high/ low KL-6 level	Median follow-up time	Survival indicators	The source of extracted HR	Hypothesis testing methods	Adjustment variables in multivariate model
Tanaka 2011 (7)	Japan	Retrospective	103	NSCLC	pT1-3, pN0-2	Curative surgery without preoperative chemotheory or radiotherapy	23/80	Not specified	PFS, OS	Multivariate Cox proportional hazards regression model	Significance test of regression coefficients (Likelihood Ratio Test)	pN stage,serum CYFRA21-1
Miyazaki 1 2010 (24)	Japan	Retrospective (consecutive)	205	LC	IA-IV	Surgery, chemotherapy, chemo-radiotherapy, irradiation or supportivecare	69/136	Not specified	OS	Multivariate Cox proportional hazards regression model	Significance test of regression coefficients	Age, gender, PS, smoking habit, pathology and stage
Miyazaki 2 2010 (24)	Japan	Retrospective (consecutive)	68	LC	IA-IV	Surgery, chemotherapy, chemo-radiotherapy, irradiation or supportivecare	50/18	Not specified	OS	Kaplan-Meier survival curves	Log rank test	None
Shoji 2016 (25)	Japan	Retrospective (consecutive)	204	NSCLC	IA	Curative surgery	69/135	50 months	PFS	Multivariate Cox proportional hazards regression model	Significance test of regression coefficients	Smoking status, histology, and intratumoral blood vessel invasion
Fujiwara 2008 (26)	Japan	Retrospective (consecutive)	41	NSCLC	IIIA-IV	Gefitinib monotherapy after failure to at least one prior chemotherapy	22/19	20.6 months	PFS, OS	Multivariate Cox proportional hazards regression model	Significance test of regression coefficients	Complicated with interstitial lung disease, PS, histology, gender, and smoking history
Ishikawa 2008 (27)	Japan	Retrospective (consecutive)	70	NSCLC	IIIB-IV and recurrence after surgery	Gefitinib monotherapy after failure to at least one prior chemotherapy	35/35	250 days	PFS, OS	Multivariate Cox proportional hazards regression model	Significance test of regression coefficients (Likelihood Ratio Test)	Sex, PS, and smoking history
Inata 2007 (8)	Japan	Retrospective	103	ADC	IA-IV	Surgery, chemotherapy, chemo-radiotherapy, radiation, or supportivecare	44/59	Not specified	OS	Multivariate Cox proportional hazards regression model	Significance test of regression coefficients	Serum KL-6/MUC1 carrying sialyl Lewisa oligosaccharide, T factor, N factor, M factor, and PS
Park 2023 (28)	South Korea	Retrospective	283	LC	I-IV	Operation, CCRT, chemotherapy, radiation, palliative treatment, or supportivecare	164/119	18.7 months	OS	Univariate Cox proportional hazards regression model	Significance test of regression coefficients	None
Kudo 2015 (29)	Japan	Prospective (consecutive)	115	NSCLC	Not specified	Gefitinib monotherapy	47/68	Not specified	OS	Multivariate Cox proportional hazards regression model	Significance test of regression coefficients	Body surface area, PS, gender, histology, staging, and pack-year
Nakahama 2023 (30)	Japan	Retrospective	202	NSCLC	III-IV or recurrent	pembrolizumab, nivolumab, or atezolizumab	78/124	Not specified	PFS, OS	Multivariate Cox proportional hazards regression model	Significance test of regression coefficients	Se, age, smoking history, PS , EGFR or ALK alteration, histological subtype, ICI treatment line, and PD-L1 expression
Han 2024 (31)	South Korea	Retrospective (consecutive)	98	NSCLC	I-IV	Surgery, radiotherapy, chemotherapy, and concurrent chemoradiation therapy	29/69	11 months	OS	Multivariate Cox proportional hazards regression model	Significance test of regression coefficients	Forced vital capacity and stage
Tomita 2016 (32)	Japan	Retrospective (consecutive)	175	NSCLC	I-III	Curative surgery	15/160	Not specified	OS	Multivariate Cox proportional hazards regression model	Significance test of regression coefficients	Gender, smoking, histology, stage, CEA, and ILD
Kikuchi 2021 (33)	Japan	Retrospective (consecutive)	56	NSCLC	III, IV or recurrence	chemotherapy	–	Not specified	OS	Univariate Cox proportional hazards regression model	Significance test of regression coefficients	None

ADC, adenocarcinoma; ALK, anaplastic lymphoma kinase; CCRT, Concurrent chemoradiation therapy; CEA, Carcinoembryonic antigen; ECLIA, electrochemiluminescence immunoassay; EGFR, epidermal growth factor receptor; ELISA, enzyme-linked immunosorbent assay; HR, hazard ratio; LC, lung cancer; MUC1, mucin 1; NSCLC, non-small cell lung cancer; PFS, progression-free survival; PS, performance status; OS, overall survival; KL-6, krebs von den Lungen-6; ICI, immune-checkpoint inhibitor; ILD, interstitial lung disease; PD-L1, programmed death-ligand 1.

**Table 2:** Assay characteristics of KL-6 across studies.

Study	Assay method	Testing equipment and/or reagents	Cut off	Method used to determine the cut-off value
Tanaka 2011 (7)	Sandwich ECLIA	Picolumi 8220 Analyzer (Sanko Junyaku, Tokyo, Japan)	400 U/mL	ROC curve analysis
Miyazaki 2010 (24)	Sandwich ELISA	A KL-6 antibody kit (Eisai,Tokyo, Japan)	500 U/mL	The levels of healthy individuals reported earlier
Shoji 2016 (25)	Not specified	–	285 U/mL	ROC curve analysis
Fujiwara 2008 (26)	ECLIA or enzyme immunoassay	–	500 U/mL	The levels of healthy individuals reported earlier
Ishikawa 2008 (27)	Sandwich ECLIA	Picolumi 8220 Analyzer (Sanko Junyaku, Tokyo, Japan)	500 U/mL	The levels of healthy individuals reported previously
Inata 2007 (8)	ELISA	EITEST KL-6 ELISA kit (Eisai, Tokyo, Japan)	500 U/mL	–
Park 2023 (28)	Immunoturbidimetric assay	AU 5800 chemistry analyzer (Beckman Coulter, Brea, CA, USA) with the Nanopia KL-6 assay (Sekisui Medical Co., Ltd., Tokyo, Japan)	302.4 U/mL	ROC curve analysis
Kudo 2015 (29)	Not specified	–	500 U/mL	–
Nakahama 2023 (30)	Not specified	–	500 U/mL	The value routinely used in Japanese clinical practice
Han 2024 (31)	Not specified	–	1000 U/mL	–
Tomita 2016 (32)	Not specified	–	500 U/mL	–
Kikuchi 2021 (33)	Not specified	–	per U/mL increment	–

ECLIA, electrochemiluminescence immunoassay; ELISA, enzyme-linked immunosorbent assay; ROC, receiver operating characteristic; KL-6, krebs von den Lungen-6.

### 3.2 KL-6 and progression-free survival

**Figure 2:**
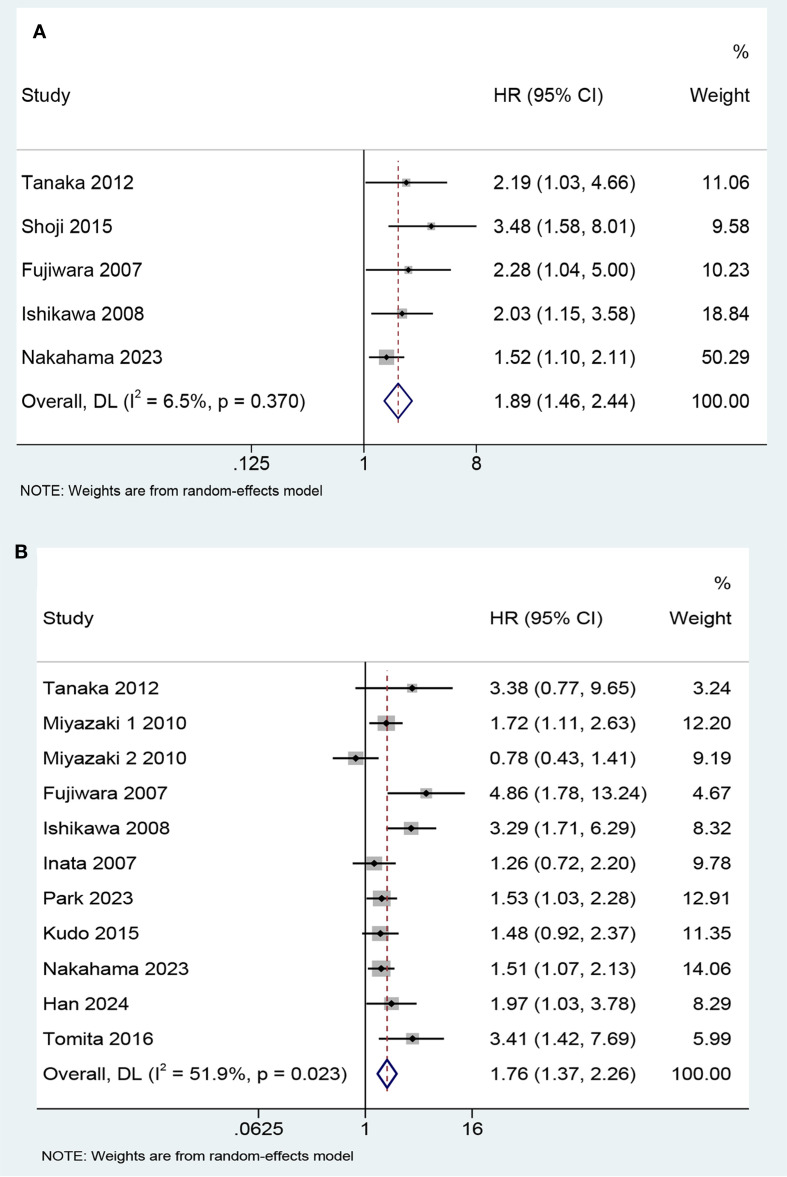
Forest plot of the predictive value of KL-6 in **(A)** progression-free survival (HR 1.89, 95%CI: 1.46-2.44, Z = 4.850, P< 0.001) and **(B)** overall survival (HR 1.76, 95%CI: 1.37-2.26, Z = 4.450, P<0.001).

**Figure 3:**
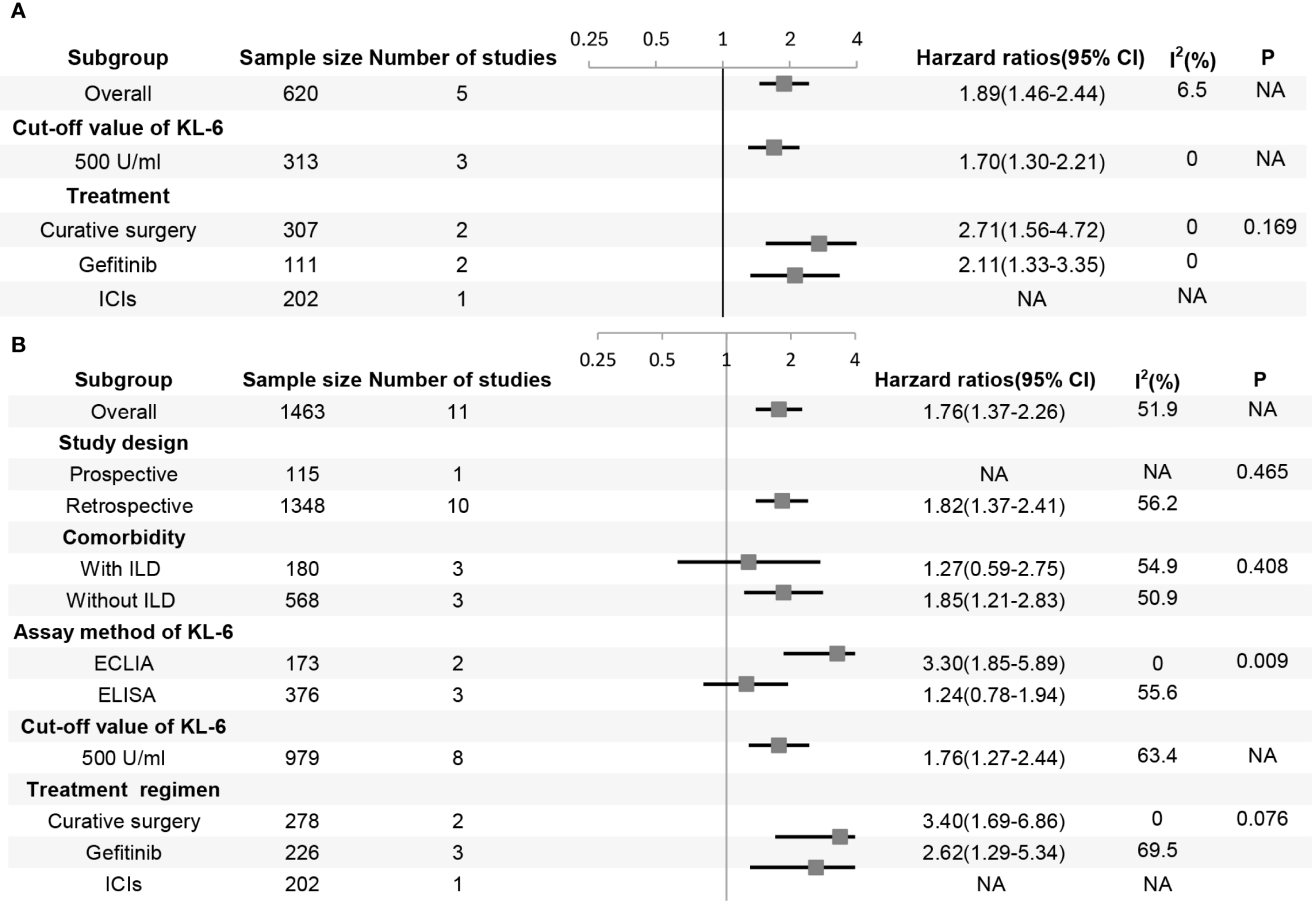
Forest plot of subgroup analysis in **(A)** progression-free survival and **(B)** overall survival. P represents the P-value for subgroup differences, ECLIA, electrochemiluminescence immunoassay; ELISA, enzyme-linked immunosorbent assay; KL-6, krebs von den Lungen-6; ICI, immune-checkpoint inhibitor; ILD, interstitial lung disease; NA, not applicable, typically for subgroups with a single study or when only one subgroup exists, and a P-value for subgroup differences is not computed.

Five studies involving 620 patients evaluated the correlation between KL-6 expression level and progression-free survival (PFS). The results showed that the patients with elevated KL-6 expression before treatment had shorter PFS than those with normal levels (HR, 1.89, 95% CI: 1.46–2.44, P < 0.001; heterogeneity: I^2^ = 6.5%, P = 0.37) (**Figure 2A**). Subgroup analysis based on treatment program revealed no significant differences between the subgroups (**Figure 3A**).

### 3.3 KL-6 and OS

Twelve studies involving 1,519 patients focused on the predictive value of KL-6 level for OS. The study by Kikuchi et al. evaluating the effect of per U/mL increment in KL-6 level on survival found that it was not a prognostic factor for NSCLC (HR 1.00, 95% CI: 0.99–1.00, P = 0.564). The remaining 11 studies statistically analyzed KL-6 level as a dichotomous variable. The pooled results indicated that higher KL-6 expression was associated with shorter OS (HR 1.76, 95% CI: 1.37–2.26, P < 0.001) (**Figure 2B**).

The results of the subgroup analyses based on studies that analyzed KL-6 as a dichotomous variable are as follows (**Figure 3B**): First, elevated KL-6 level demonstrated a stronger association with shorter OS in studies using electrochemiluminescence immunoassay (ECLIA) than in studies using ELISA (enzyme-linked immunosorbent assay) methods (HR 3.30, 95% CI: 1.85–5.89 vs HR 1.24, 95% CI: 0.78–1.94, P = 0.009). Second, patients with lung cancer may have ILD. The treatment of tumors can also cause interstitial lung injury, such as radiation pneumonitis and immunotherapy-related lung injury. Therefore, the patients were stratified according to the presence or absence of ILD. The prognostic value of elevated KL-6 for OS was significant for patients with lung cancer without ILD (HR 1.85, 95% CI: 1.21–2.83; P = 0.005) but not for those with ILD (HR 1.27, 95% CI: 0.59–2.75; P = 0.539). Third, most studies used 500 U/mL as the cut-off value for KL-6 level. The prognosis of patients worsened when KL-6 level was higher than 500 U/mL before treatment. The subgroup analyses of the study design and treatment protocols showed no significant differences.

### 3.4 Heterogeneity analysis and sensitivity analysis

**Figure 4:**
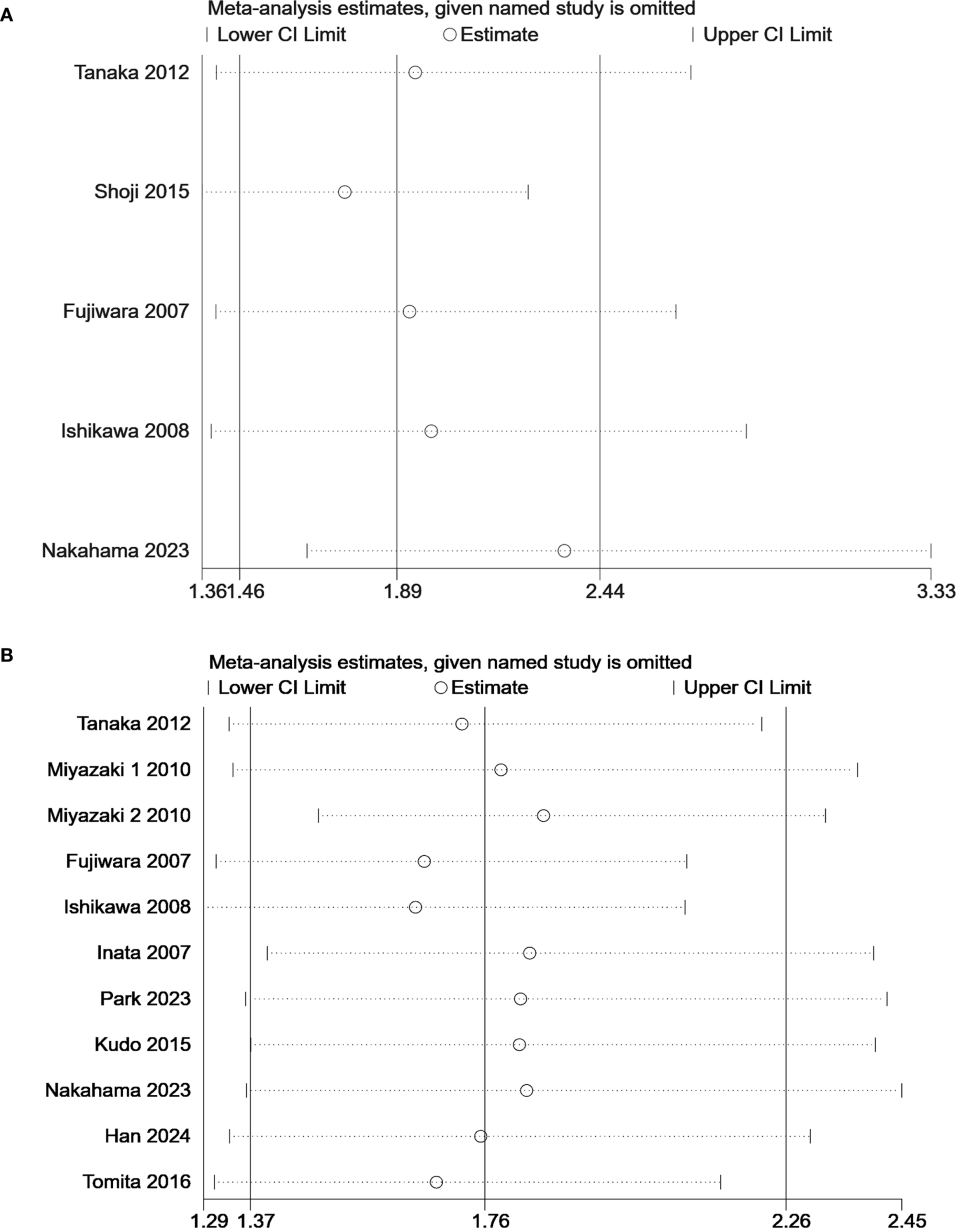
Sensitivity analysis of the predictive value KL-6 in **(A)** progression-free survival and **(B)** overall survival.

Statistically significant heterogeneity was found in OS (I²=51.9%, P = 0.023) (**Figure 2B**). The results of subgroup analysis showed that the heterogeneity of each subgroup did not yield a significant reduction (**Figure 3B**). A Galbraith plot was generated to graphically identify inter-study heterogeneity. The results of the studies by Miyazaki 2 (24), Fujiwara (26), and Ishikawa (27) were outliers in the Galbraith plot (**Supplementary Figure S1**). The inter-study heterogeneity was significantly reduced after the removal of these three studies (I²=0%, P = 0.555), and the pooled result was not significantly affected (HR 1.63, 95% CI: 1.36–1.94, P<0.001) (**Supplementary Figure S2**). The leave-one-out sensitivity analyses also indicated that no single study significantly affected the pooled results (**Figure 4**). These results confirmed the robustness of our findings.

### 3.5 Publication bias

**Figure 5:**
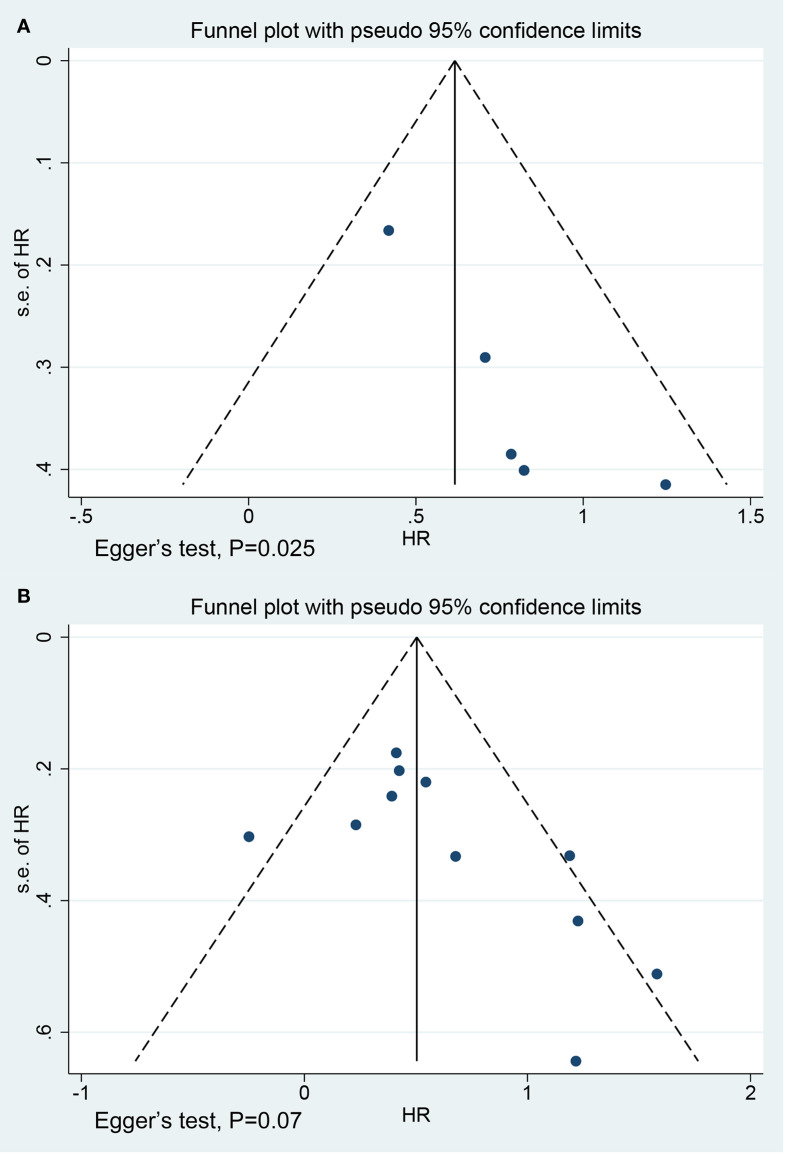
Funnel plots for studies on **(A)** progression-free survival (Egger’s test, P = 0.025) and **(B)** overall survival (Egger’s test, P = 0.07).

No significant publication bias was observed in the studies on OS (P = 0.07) (**Figure 5B**). However, the funnel plots for the studies on PFS (P = 0.025) were asymmetric (**Figure 5A**). We further generated a contour-enhanced funnel plot using the trim and fill method to adjust for potential publication bias and assess the robustness of the results. The results suggested that publication bias may lead to the asymmetry of the funnel plot for PFS (**Supplementary Figure S3**). However, the results remained unchanged after the addition of three hypothetically missing studies (**Supplementary Table S3**).

## 4 Discussion

Previous meta-analyses have reported the predictive role of serum cytokeratin 19 fragment concentrations for the prognosis of lung cancer based on serological biomarkers, but only for NSCLC (34). Another study confirmed the predictive value of MUC1 for NSCLC (35). The previous meta-analysis included studies detecting MUC1 in tumor tissue, mRNA concentrations of MUC1 in blood, and serum KL-6. However, it did not explore the predictive value of KL-6 level. The serum KL-6 was used as the main analytical index to explore its predictive value for lung cancer (including small cell lung cancer and NSCLC). The serum KL-6 level can predict the prognosis of lung cancer without ILD in the Asian population. This finding has not been previously reported in the meta-analysis. Our research focused on the predictive value of serum KL-6 before treatment, which is simple and readily obtainable. This could enable clinicians to make early assessments of tumor progression in patients, facilitate the formulation of personalized therapy, and reduce the risk of cancer-related mortality.

MUC1, an oncogenic molecule, promotes the occurrence, development, and metastasis of lung cancer through multiple mechanisms (36–38). Targeting MUC1 inhibits programmed cell death ligand 1 expression (39), suppresses epidermal growth factor receptor activation (40), and enhances CD8+ T cell infiltration and antitumor activity (41). MUC1 has been identified as a promising therapeutic target and a clinically relevant biomarker in cancer treatment. KL-6 is a specific glycoform of MUC1 that is recognized by a murine monoclonal antibody. Tang et al. (42) demonstrated significant associations of positive KL-6 expression with lymph node metastasis, tumor invasion, and advanced tumor stage in pancreatic cancer. The 5-year survival rate of patients with positive KL-6 expression was significantly lower than that of patients without KL-6 expression. Xu et al. (43) further confirmed that KL-6 overexpression may induce tumor metastasis by inhibiting the expressions of E-cadherin and β-catenin proteins. The relationships between serum KL-6 levels before treatment and the prognosis of lung cancer have not been reported. We confirmed serum KL-6 levels before treatment as a predictor of lung cancer prognosis and recommended the detection method and cut-off value for this purpose. KL-6 is produced by epithelial cells during alveolar damage and regeneration. However, the source of circulating KL-6 in patients with lung cancer is still under discussion. Moriyama et al. (44) reported that the serum KL-6 levels in patients with liver cancer were positively correlated with tumor size and decreased after liver cancer treatment. They found KL-6 antigen expression in the cell membrane and endoplasmic reticulum of hepatocellular carcinoma cells via immunoelectron microscopy. They consequently presumed that the KL-6 antigen was produced by cancer cells. Tanaka et al. (7) reported correlations between the depolarized expression patterns of KL-6 in lung cancer tissues and preoperative circulating KL-6 levels. The KL-6 levels in the blood of patients with lung cancer decreased significantly after surgery. Ishikawa et al. (27) also reported that the serum KL-6 levels of patients who responded well to gefitinib treatment gradually and significantly declined after 2 and 4 weeks of treatment. These findings suggested that serum KL-6 levels in patients with lung cancer are derived from the primary tumor.

The results of the subgroup analysis showed that the KL-6 level determined using the ECLIA test was more predictive of OS for lung cancer than that determined using the ELISA method. The original data for KL-6 were not provided in the included studies. Therefore, we were unable to perform direct comparisons or consistency analyses between the two detection methods. No study has reported direct comparisons of ELISA and ECLIA for KL-6 measurement. However, previous studies have reported that ECLIA has superior diagnostic sensitivity, specificity, and concordance rates to those of ELISA in detecting viral antibodies (45–47). The better predictive value may be attributed to the higher sensitivity and better linear range of the ECLIA. Further studies are needed to directly compare the two detection methods for KL-6 measurement.

Most of the included studies used a cut-off value of 500 U/mL for KL-6 level, whereas Han et al. (31), Tanaka et al. (7), and Park et al. (28) used cut-off values of 1000, 400, and 302.4 U/mL, respectively. Therefore, subgroup analysis stratified by different cut-off values was not feasible in the current study. We pooled the results of studies that set the KL-6 threshold at 500 U/mL and found that the prognosis of patients with KL-6 levels greater than 500 U/mL was poorer. Further studies are needed to explore the optimal cut-off value of KL-6 based on receiver operating characteristic curve analysis.

Serum KL-6 levels were elevated in patients with ILD relative to those without; this was especially observed among patients with ILD who had disease progression and poor prognosis (9, 48). Fathi et al. (49) reported negative correlations between serum KL-6 levels and the percentages of pulmonary function test parameters (such as total lung capacity, forced vital capacity, and carbon monoxide diffusion capacity) for patients with ILD. Tanaka et al. (50) further confirmed that the annual change rate of KL-6 was an independent prognostic factor for acute exacerbation of ILD. These results suggest that KL-6 is associated with the severity of ILD and the risk of death. ILD is a common comorbidity of lung cancer with an incidence of 2.4–10.9% (51). The presence of ILD is a major independent negative prognostic factor in patients with lung cancer. It is associated with increased mortality and treatment complications (52, 53). The effects of lung cancer combined with ILD on the prognostic predictive function of KL-6 remain unclear. Our results showed that elevated serum KL-6 levels were significantly associated with worse OS of patients with lung cancer without ILD but not in those with ILD. Therefore, an elevated KL-6 level in a patient with lung cancer and ILD is likely to reflect the severity and activity of the underlying fibrotic lung disease rather than provide independent prognostic information about the cancer. Interestingly, Miyazaki et al. (24) and Tomita et al. (32) did not observe the predictive effect of serum KL-6 levels greater than 500 U/mL for poor prognoses of lung cancer with concurrent ILD. Han et al. (31) set the cut-off value for elevated KL-6 to 1000 U/mL and showed that increased KL-6 level predicts worse OS in patients with lung cancer and ILD. Higher KL-6 levels may carry a cancer-specific prognostic signal that can be detected above the high-level “noise” generated by the underlying ILD. The severity of ILD (based on indicators such as pulmonary function testing parameters and high-resolution CT fibrosis scores) must be adjusted to assess the cancer-specific predictive value of KL-6 for lung cancer combined with ILD. Large-scale prospective studies are required to further validate this hypothesis and establish and clinically validate a population-specific KL-6 level cut-off based on receiver operating characteristic curve analysis for this complex patient population.

Park et al. (28) reported that elevated KL-6 level before treatment was an independent risk factor for the development of severe treatment-related ILD (TR-ILD) (n=26). TR-ILD is a potentially fatal complication of lung cancer therapy, this finding has significant clinical implications for optimizing treatment strategies in patients with elevated baseline KL-6 levels. However, Kawase et al. (54) reported the findings of a retrospective analysis of 341 patients with NSCLC who were treated with epidermal growth factor receptor-tyrosine kinase inhibitors. They found that the baseline KL-6 levels neither predicted epidermal growth factor receptor-tyrosine kinase inhibitor-related ILD development nor differentiated between fatal and non-fatal ILD cases. Evidence regarding this association is limited. Further prospective studies are warranted to validate the predictive role of baseline KL-6 levels for TR-ILD.

We explored potential sources of variation to ensure the reliability of the findings based on the significant heterogeneity observed in the OS results. Subgroup analyses stratified by comorbidities, KL-6 detection methodologies, and lung cancer treatment regimens failed to identify significant sources of heterogeneity. The Galbraith plot showed that the studies by Miyazaki 2 (24), Fujiwara (26), and Ishikawa (27) are the main sources of inter-study heterogeneity. Park et al. (28) reported higher serum KL-6 levels in patients with adenocarcinoma than in those with squamous cell carcinoma and small cell carcinoma. Nakahama et al. (30) and Miyazaki et al. (24) also reported a higher proportion of elevated KL-6 levels in patients with adenocarcinoma than in those with other histological subtypes. These results suggest potential tumor-type specificity of KL-6 expression in lung cancer. The three outlier studies identified in the Galbraith plot had significantly higher proportions of adenocarcinoma patients than the other studies (mean 71.5% vs mean 61.5%). This suggests that differences in tumor histology may represent one potential source of heterogeneity; however, insufficient histologically related data reporting in the included studies prevented further subgroup analyses to confirm this hypothesis. Furthermore, several critical details that also likely contributed to the observed between-study heterogeneity were not reported in some studies, including follow-up duration, ILD diagnostic criteria, KL-6 detection kits, and cut-off value determination methods. These reporting gaps limited our ability to conduct a more in-depth exploration.

Some limitations of our study should be highlighted. First, most of the enrolled studies were retrospective. However, the quality assessments of the included studies were acceptable, and the pooled results were positive. Prospective cohort studies are needed for clinical verification in the future. Second, the samples in the study were all from Asian individuals, and the recommendation of a cut-off value of 500 U/mL was derived exclusively from cohorts of Asian individuals. Accumulating evidence has demonstrated significant ethnic variations in KL-6/MUC1 expression levels (55–57), which are primarily mediated by MUC1 gene polymorphisms (58). Gad et al. (59) confirmed that serum KL-6 levels were significantly higher in Egyptian patients with hepatocellular carcinoma than in Japanese patients. The predictive value of KL-6 levels for lung cancer in other races needs further exploration. Third, more than 90% of the participants in this study had NSCLC. Future studies should investigate the predictive value of KL-6 levels in patients with small cell lung cancer. Fourth, the trim-and-fill analysis demonstrated robust results after filling in the missing studies, suggesting that the potential publication bias associated with studies investigating the prognostic value of KL-6 for PFS has a limited impact on the overall findings. Fifth, the HR in the study by Park et al. (28) was extracted from the univariate analysis, which provided weaker evidence due to the lack of control for confounding factors. However, the sensitivity analysis confirmed the robustness of our findings.

The elevated KL-6 level before treatment was a biomarker of poor prognosis of patients with lung cancer without ILD in the Asian population. ECLIA may be a more sensitive method for detecting the prognostic value of KL-6. This hypothesis warrants confirmation in direct head-to-head comparative studies. The cut-off value of approximately 500 U/mL is frequently used and associated with poor prognosis in Asian patients with lung cancer. However, it requires rigorous validation in large, prospective, multi-ethnic cohorts before it can be considered for broad clinical application, especially given the known genetic variations that affect baseline KL-6 levels. For patients with lung cancer and ILD, serum KL-6 levels >500 U/mL is more likely to indicate the severity and activity of the underlying fibrotic lung disease rather than providing independent prognostic information about the cancer itself, further investigation is still required to validate that whether KL-6 is a cancer-specific predictive biomarker for these population and what is an optimal cut-off value.

## Data Availability

The original contributions presented in the study are included in the article/**Supplementary Material**. Further inquiries can be directed to the corresponding author.
