# Supplementary material for: Serum Krebs von den Lungen-6 before treatment predicts the prognosis of lung cancer in Asian populations: a systematic review and meta-analysis
Source: Front Immunol. 2025 Sep 17;16:1644573. doi: 10.3389/fimmu.2025.1644573 (PMC12484158; doi:10.3389/fimmu.2025.1644573)
Supplement: Supplementary file 1 [file DataSheet1.pdf]

Supplementary Material

1 Supplementary Figures

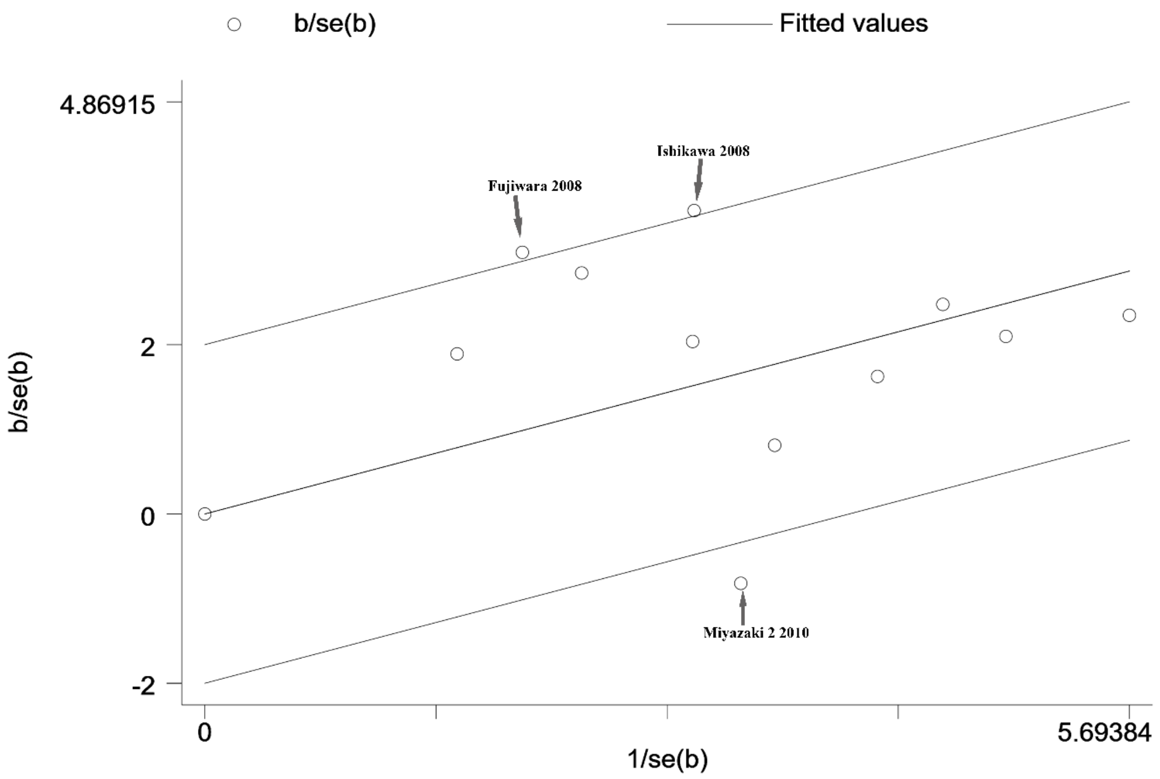

Figure S1 Galbraith plot of the predictive value of KL-6 in overall survival

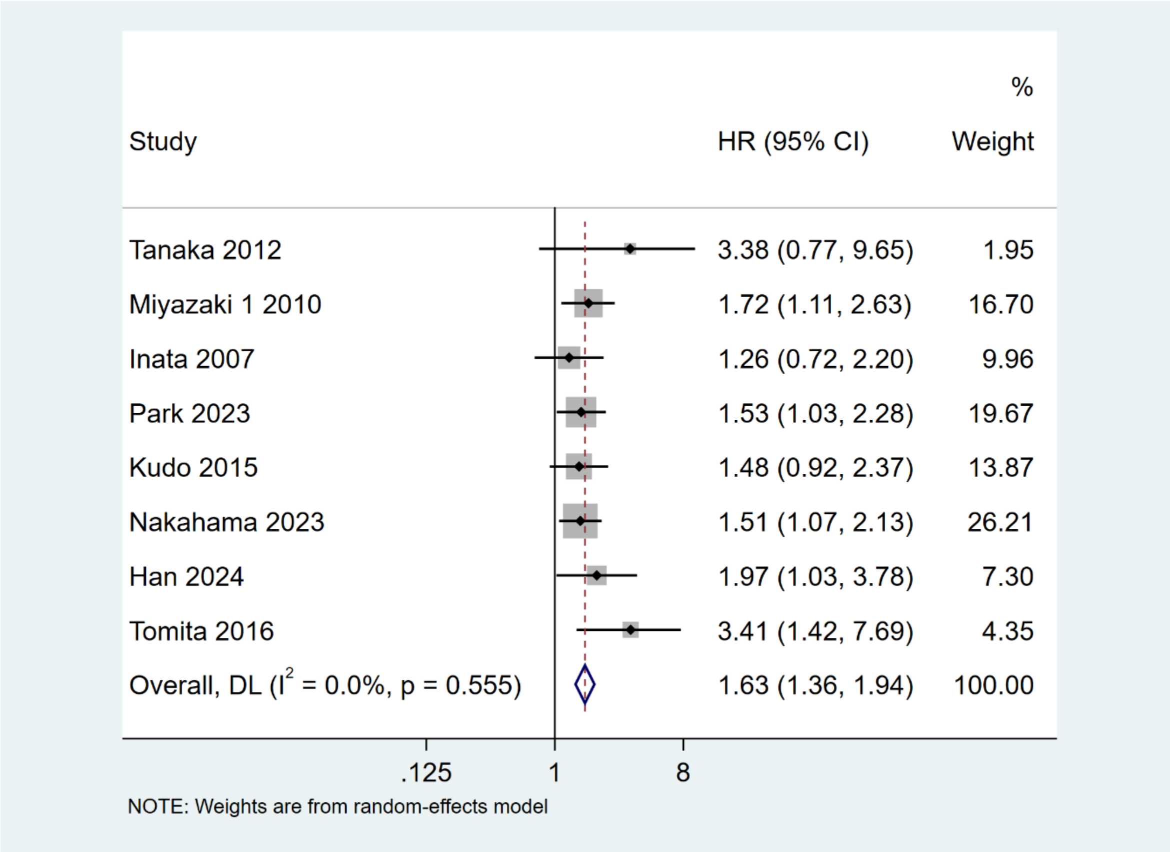

Figure S2 Forest plot of the predictive value of KL-6 in overall survival after deleting the Miyazaki 2 2010 study, Fujiwara 2008 study, and Ishikawa 2008 study

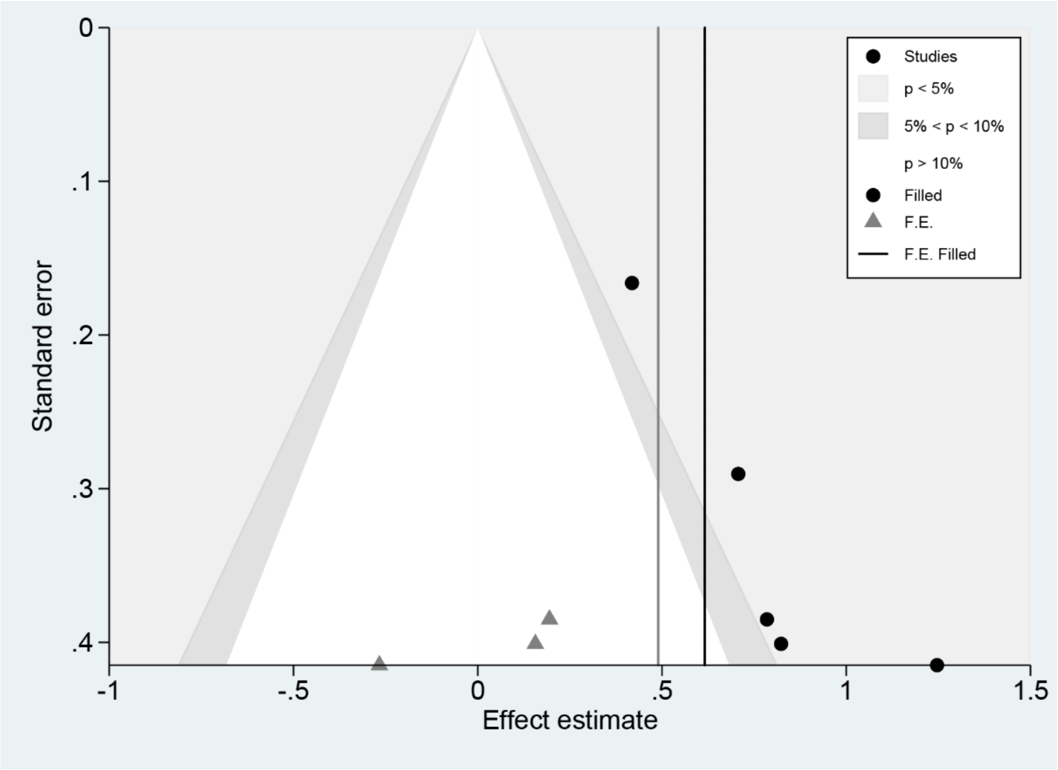

Figure S3 Contour-enhanced funnel plot with the trim and fill method of studies on the predictive value of KL-6 in progression-free survival

2 Supplementary Tables

Table S1: Literature search strategy (Search date: June 23, 2025)

**Database**	**key terms and the queries**
Pubmed (n=232)	(("lung cancer"[Title/Abstract]) OR ("lung carcinoma"[Title/Abstract])) AND ((((KL-6[Title/Abstract]) OR ("Krebs von den Lungen-6"[Title/Abstract])) OR ("MUC-1"[Title/Abstract])) OR (mucin-1[Title/Abstract]))
Embase(n=1044)	#1 'kl-6' AND [abstracts]/lim<br>#2 'krebs von den lungen-6' AND [abstracts]/lim<br>#3 'muc 1' AND [abstracts]/lim<br>#4 'mucin-1' AND [abstracts]/lim<br>#5 #1 OR #2 OR #3 OR #4

	#6 'lung cancer' AND [abstracts]/lim<br><br>#7 'lung carcinoma' AND [abstracts]/lim<br><br>#8 #6 OR #7<br><br>#9 #5 AND #8
Cochranelibrary (n=24)	#1 ("kl-6"):ti,kb,tw OR ("krebs von den lungen-6") :ti,kb,tw OR (MUC-1) :ti,kb,tw OR (mucin-1) :ti,kb,tw<br><br>#2 ("lung cancer") :ti,kb,tw OR ("lung carcinoma") :ti,kb,tw<br><br>#3 #1 AND #2
Web of Science (n=434)	#1 (AB=KL-6) OR (AB="Krebs von den Lungen-6") OR(AB= MUC-1) OR (AB=mucin-1)<br><br>#2 (AB=("lung cancer")) OR AB=("lung carcinoma")<br><br>#3 #1 AND #2

Table S2 Risk of bias assessment

Study	Study Participation	Study Attrition	Prognostic Factor Measurement	Outcome Measurement	Study Confounding	Statistical analysis and reporting	Overall assessment
Tanaka 2011	M	M	L	L	L	M	L
Miyazaki 2010	M	M	M	M	L	M	M
Shoji 2016	L	M	M	L	L	M	L
Fujiwara 2008	L	L	M	L	L	M	L
Ishikawa 2008	M	L	L	L	M	H	L

Inata 2007	M	L	L	L	M	M	L
Park 2023	M	H	M	L	M	M	M
Kudo 2015	L	M	M	L	M	M	M
Nakahama 2023	M	L	M	L	L	M	L
Han 2024	M	L	M	M	L	M	M
Tomita 2016	L	L	M	M	M	M	M
Kikuchi 2021	L	L	M	L	M	M	L

Abbreviation: H: High; L: Low; M: Moderate;

Table S3. Results of the trim-and-fill method

	Model	Missing studies	Before, HR (95%CI)	After, HR (95%CI)
**PFS**	Random effect	3	1.89(1.46-2.44)	1.66(1.26-2.18)

Abbreviation: HR, hazard ratio; PFS, progression-free survival.
